# Bone resorption during the first year after implantation of a single-segment dynamic interspinous stabilization device and its risk factors

**DOI:** 10.1186/s12891-015-0561-y

**Published:** 2015-05-14

**Authors:** Kaifeng Wang, Zhenqi Zhu, Bo Wang, Yi Zhu, Haiying Liu

**Affiliations:** Department of Spinal Surgery, Peking University People’s Hospital, 11 Xizhimen South Street, West District, Beijing, China

**Keywords:** Bone resorption, Risk factor, Interspinous dynamic stabilization device, Wallis

## Abstract

**Background:**

Dynamic interspinous stabilization devices generally provide satisfactory results, but can result in recurrent lumbar disc herniation, spinous process fracture, or bone resorption of the spinous process. The purpose of this study was to investigate if the Wallis dynamic stabilization device is associated with bone resorption.

**Methods:**

Patients who underwent single-segment posterior lumbar decompression and implantation of a Wallis dynamic interspinous stabilization device at the L4/5 level between January 1, 2009 and October 1, 2011 were included. Bone resorption rate, Oswestry Disability Index (ODI), Japanese Orthopedic Association (JOA) score, and visual analogue scale (VAS) pain score were measured. Patient baseline and 1-year follow-up data were collected and analyzed. The bone resorption rate of the L4 and L5 spinous processes was calculated.

**Results:**

Twenty four males and 20 females with a mean age of 42.7 ± 14.7 years were included. Twenty nine patients had significant bone resorption (bone resorption rate > 20%) and 15 had no bone resorption (bone resorption rate ≤ 20%) at 1 year after surgery. Lumbar lordosis ≥ 50° was associated with a lower bone resorption than lumbar lordosis < 50° and increasing BMI was associated with increased bone resorption. There were no significant differences between the bone resorption and no bone resorption groups in the improvement rate of VAS pain score, ODI, and JOA score at 1 year after surgery.

**Conclusions:**

Significant bone resorption occurs within 1 year after implantation of the Wallis device in more than 50% of patients. However, it does not affect short-term functional results.

## Background

Spinal fusion is the treatment of choice for a number of lumbar diseases, and has advantages such as predictable outcomes, low recurrence rate, and high lumbar spine stability. However, complications can include decreased lumbar spine motion and adjacent segment degeneration (ASD) [1]. ASD may lead to lumbar spine instability, increased facet joint stress, and subsequent symptoms such as lower back and radicular pain [2-4]. To avoid the occurrence of ASD and the pain caused by lumbar spine instability, Senegas et al. [5] introduced a dynamic interspinous stabilization device which provides non-rigid fixation. Later, a second generation implant (Wallis) was introduced made of polyetheretherketone (PEEK) which reduces, but does not prevent, motion of the segment and lowers stress in the annulus and disc fibers [6,7]. Proposed indications for the Wallis system include discectomy for massive herniated disc leading to substantial loss of disc material, a second discectomy for recurrence of herniated disc, discectomy for herniation of a transitional disc with sacralization of L5, degenerative disc disease at a level adjacent to a previous fusion, and isolated Modic I lesion leading to chronic low-back pain [6].

A long-term follow-up study including 142 patients showed that the Wallis device provided satisfactory outcomes and could prevent ASD by preserving the segmental activity [8]. A subsequent study of the same population at a mean follow-up of 13 years showed good outcomes [7]. Korovessis et al [9] carried out a 5-year follow-up study and found that the Wallis device changed the natural disease course of ASD, reduced the incidence of ASD, and avoided fusion cephalic segment. Liu et al. [10] further studied the technique and reported that the Wallis device combining with posterior lumbar interbody fusion (PLIF; topping-off) could treat mild to moderate adjacent segment degeneration, improve related symptoms, prevent the occurrence of ASD, and preserve the adjacent segment flexion and extension.

It has been reported, however, that the dynamic interspinous stabilization can result in complications including recurrent lumbar disc herniation, spinous process fracture, or even bone resorption of the spinous process. Floman et al. [11] studied 37 patients who were implanted with the Wallis device at L4-5 and followed-up for a mean duration of 16 months, and found that 13% of the patients had recurrent lumbar disc herniation at the treated segment. Wilke et al. [12] reported that though the Wallis device could reduce disc loading in the lumbar spine during extension, it resulted in minimal change of disc loading during flexion, lateral bending, and rotation, indicating that the device could not effectively distribute the load of the lumbar intervertebral disc.

With continuous development of the interspinous process spacers (IPSs), their limitations and complications have been recognized. Kim et al. [13] reported a significant correlation between degenerative spondylolisthesis and spinous process fracture following IPS surgery, and the incidence of lumbar spondylolisthesis was higher in patients with osteoporosis. Case reports have shown stress fractures in the bilateral facet joint and bone resorption of the spinous process after IPS surgery [14,15].

It is not clear why dynamic interspinous internal fixation may lead to the aforementioned complications. It is also unclear if the devices alter the structure of the spinous processes. A search of PubMed revealed more than 600 articles regarding spine surgery and bone resorption, but there were no studies examining the Wallis device and bone resorption. Thus, the purpose of this study was to investigate if implantation of the Wallis device is associated with bone resorption, and if so, what factors affect the occurrence of bone resorption and functional outcomes. We hypothesized that pressure of the Wallis device against the spinous processes may contribute bone resorption.

## Methods

### Patients

Patients between 16 and 70 years of age who underwent single-segment posterior lumbar decompression and implantation of a Wallis dynamic interspinous stabilization device at the L4/5 level between January 1, 2009 and October 1, 2011 at our institution were included in the study. All patients had moderate to severe lower extremity pain or numbness with or without lower back pain, and intermittent claudication before surgery. Symptoms were aggravated after standing or walking, and relieved after lying or flexion. All patients had received a minimum of 6 months of conservative therapy including pain medications, massage, and/or epidural cortical steroid injections without relief. In all cases, preoperative magnetic resonance imaging (MRI) confirmed L4/5 disc herniation with or without a decreased disc signal in the T2 weighted image (“black disc” change), or radiographic lumbar spinal stenosis. X-ray examination showed disc degeneration equal to or less than UCLA grade II [16].

The Meyerding classification was used to quantify the degree of spondylolisthesis [17]. Briefly, grade I is 0-25% slip, grade II is 26-50% slip, grade III is 51-75% slip, and grade IV is 75-99% slip. Grade V is complete slip (100%). Only patients with grade I spondylolisthesis were included in the study. Patients with more than 10° (Cobb angles) of lumbar scoliosis, osteoporosis (T-score < -2.5 [18]), the presence of vertebral fractures caused by osteoporosis, and those with a loss of motion in the surgical segment were excluded. This study was approved by the Institutional Review Board of Peking University People’s Hospital, Beijing, China, and all patients provided written informed consent for the surgical procedures performed.

### Surgical method

All surgeries were performed by a professional and experienced spinal surgeon with more than 25 years of experience performing spine surgery and more than 10 years of experience with the Wallis device.

The patient was placed in the prone position, general anesthesia was induced, the surgical area was prepared, and a longitudinal midline incision about 6 cm in length was made in the lower back. The skin and superficial fascia were incised, and the lumbodorsal fascia was dissected. The L4/5 supraspinous ligament was protected, and the paraspinal muscles were dissected to expose the L4 and L5 spinous processes, laminae, and facets. After confirming acceptable stability of L4/5, the interspinous ligament between the L4 and L5 spinous processes was removed. The upper margin of the L5 lamina, the lower margin of the L4 lamina, and the medial osteophyte of the L4/5 facet were removed. The L4/5 ligamentum flavum was released sufficiently and removed, and bilateral L5 nerve roots were released. The L4/5 nucleus pulposus was removed under direct visualization, and the L4/5 intervertebral space was cleaned. Satisfactory release and excellent mobility of the bilateral L5 nerve roots were confirmed. Gelatin sponge was used for hemostasis within the spinal canal.

The L4 and L5 spinous processes were trimmed, and an elastic interspinous internal fixation device (Wallis) of a suitable size was inserted and fixed to the L4 and L5 spinous processes. The stability of the L4/5 segment was checked, and wound irrigation was performed. A drainage tube was placed in the surgical field. The L4/5 supraspinous ligament was sutured to corresponding spinous processes. Layered closure was then performed.

### Radiographic examinations and measurements

**Figure 1:**
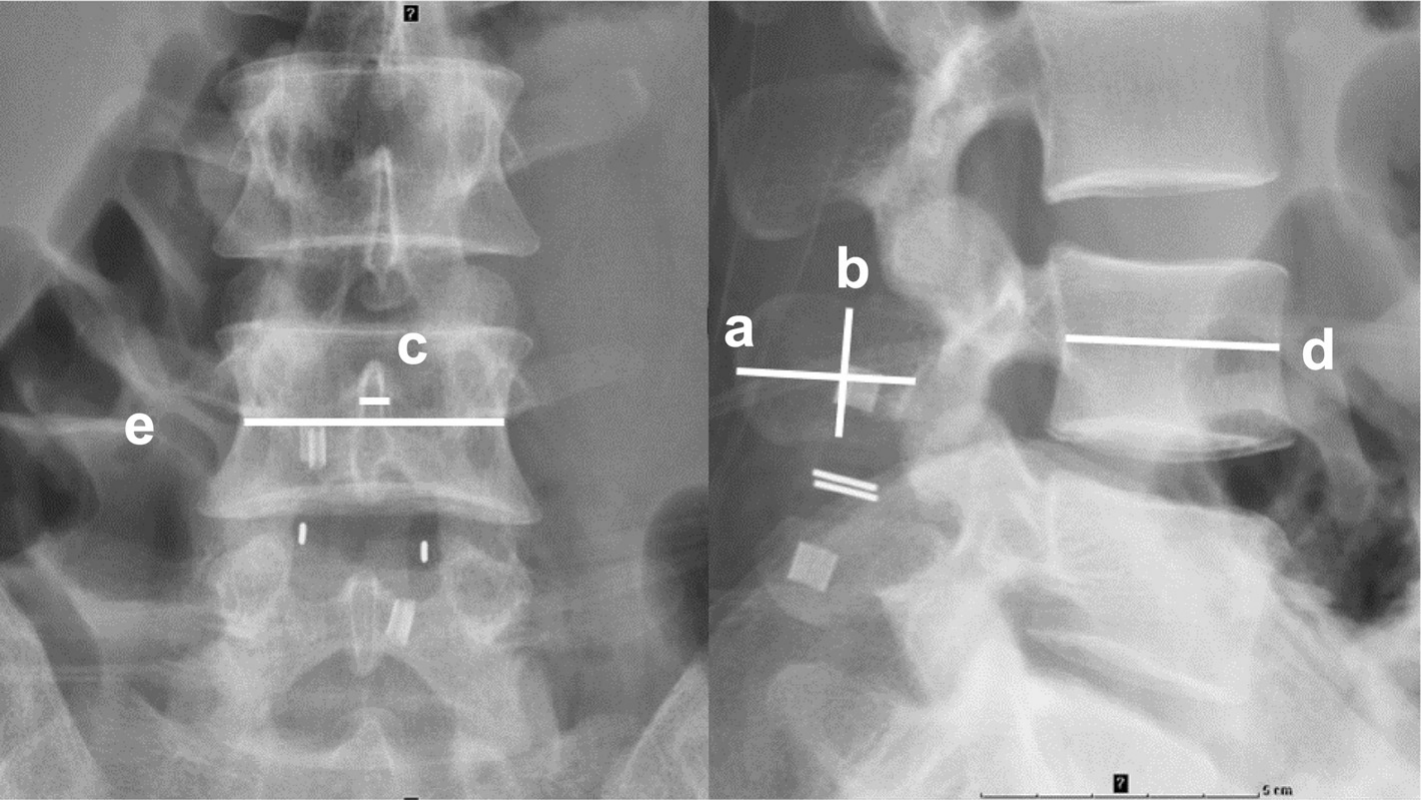
**Radiographic measurement.** Right: Later view. Left: Anteroposterior view. **a**=Length of spinous process. **b**=Height of spinous process. **c**=Width of spinous process. **d**=Length of the L4 vertebrae. **e**=Width of the L4 vertebrae.

Anteroposterior and lateral lumbar spine radiographs in the neutral position were obtained before surgery, and 1 week and 1 year after surgery. Lateral flexion and extension radiographs were obtained before surgery and 1 year after surgery. During the dynamic lumbar X-ray examinations, the patients were required to flex and extend the lumbar spine as much as possible without causing discomfort. Radiographic measurements performed are illustrated in Figure 1, and have been described in prior studies [10,19]. Radiographic measurements of the L4 and L5 spinous processes included: length of the spinous process (A: the distance from the midpoint of the spinous process base to the apex of the spinous process); height of the spinous process (B: the distance between the midpoint of the upper margin of the lower spinous process and that of the lower margin of the upper spinous process, namely the space for Wallis device insertion); thickness of the spinous process (C: the thickness of the middle part of the spinous process in the anteroposterior view); sagittal diameter of the L4 vertebral body on the lateral view (D); width of the L4 vertebral body on the anteroposterior view (E); distance between the L4 and L5 spinous processes in the neutral position (F: half the sum of the distance between anterior margins and the distance between posterior margins of the spinous process); height of the intervertebral space (G: half the sum of the distance between anterior margins and the distance between posterior margins of the intervertebral space); overall lumbar lordotic angle (LL: the angle between the upper endplate of the L1 vertebral body and the upper endplate of the S1 vertebral body).

To avoid possible magnification error in the radiographic examinations, the relative length of the spinous process (a = A/D × 100), relative height of the spinous process (b = B/D × 100), relative thickness of the spinous process (c = C/E × 100), relative distance between 2 spinous processes (f = F/D × 100), relative height of the intervertebral space (g = G/D × 100), and rate of bone resorption (H = [value measured 1 week after surgery – the value measured 1 year after surgery]/value measured 1 year after surgery × 100) were used as evaluation measures [19,20]. All measurements were made on digital images displayed on the picture archiving and communication system (PACS). Centricity RIS CE V3.0 was used for recording the lines, and the measured data were obtained from the computer automatically.

All patients received an MRI 1 year after surgery. Degeneration in the L3/4, L4/5, and L5/S1 discs were graded according to the Pfirrmann grading system [21].

### Outcome measures

All patients were followed-up for a minimum of 1 year, and patient data collected included age, sex, height, weight, body mass index (BMI), smoking status, preoperative presence of osteoporosis, operative time, intraoperative blood loss, duration of postoperative brace application, and duration of postoperative active back muscle exercise. Oswestry Disability Index (ODI), Japanese Orthopedic Association (JOA) scores, and visual analogue scale (VAS) pain scores were measured before surgery, 1 week after surgery, and 1 year after surgery. The rate of symptom improvement was calculated as follows: the rate of ODI or VAS improvement = (preoperative score – postoperative score)/preoperative score; the rate of JOA improvement = (postoperative score – preoperative score)/postoperative score.

Bone resorption was based on the length, height, and thickness of the L4 and L5 spinous processes. Bone resorption was defined as a decrease of any parameter by an amount of 20% or more. Bone resorption rate = (value at 1 week postoperative – value at 1 year postoperative)/value at 1 year postoperative × 100. Significant bone resorption defined as a bone resorption rate > 20%. Patients were divided into 2 groups: bone resorption group (bone resorption rate > 20%) and no bone resorption group (bone resorption rate ≤ 20%).

### Statistical analysis

Normally distributed continuous data were presented as mean and standard deviation, and compared with Student’s *t* test between 2 groups. Non-normally distributed continuous data were presented as median and inter-quartile range (IQR, the range between the 25th and 75th percentile), and compared with the Mann-Whitney *U* test between 2 groups. Categorical variables were presented as count and percentage, and compared by the Chi-square test or Fisher’s exact test as appropriate. To identify risk factors associated with bone resorption, the point estimates and 95% confidence intervals (CIs) of odds ratios (ORs) were calculated by univariate logistic regression models. Variables were included in the multivariate logistic regression model if they reached significance in the univariate analysis (p < .01). All statistic assessments were evaluated at a 2 sided α level of 0.05. Statistical analyses were performed by using SAS software package, version 9.2 (SAS Institute Inc., Cary, NC, USA).

## Results

A total of 44 patients, 24 males and 20 females, with a mean age of 42.7 ± 14.7 years, who received surgery between January 2009 and October 2011, and had complete follow-up data were included in the analysis. The patients were divided into a bone resorption (bone resorption rate > 20%) (n = 29) group and a no bone resorption (bone resorption rate ≤ 20%) (n = 15) group based on the bone resorption rate at 1 year after surgery. The baseline demographic and clinical data of the two groups are presented in Table 1. The distribution of lumbar lordosis was significantly different between bone resorption and no bone resorption groups (p = .007, Table 1). There were no significant differences in intraoperative or postoperative parameters between the two groups (Table 2).

**Table 1:** **Patient demographic and clinical data**

	**Bone resorption (n = 29)**	**Nobone resorption (n = 15)**	**p**
Age, y	42.8 ± 15.0	42.4 ± 14.5	.928
Gender			.139
Female	16 (55.2)	4 (26.7)	
Male	13 (44.8)	11 (73.3)	
BMI, kg/m^2^	25.7 ± 2.7	23.5 ± 4.5	.097
Smoking			.260
Never smoked	21 (72.4)	8 (53.3)	
Light smokers	5 (17.2)	3 (20.0)	
Severe smokers	3 (10.3)	4 (26.7)	
Osteoporosis	7 (24.1)	0 (0.0)	.077
Lumbar lordosis,	39.19 ± 13.85	44.53 ± 15.15	.248
<50	24 (82.8)	6 (40.0)	.007*
≥50	5 (17.2)	9 (60.0)	
L4-5 spinous height			.368
≤20	14 (48.3)	6 (40.0)	
20-30	6 (20.7)	1 (6.7)	
30-40	5 (17.2)	6 (40.0)	
≥40	4 (13.8)	2 (13.3)	
L4-5 Interspinous height			.775
≤25	7 (24.1)	2 (13.3)	
25-30	8 (27.6)	6 (40.0)	
30-35	11 (37.9)	6 (40.0)	
≥35	3 (10.3)	1 (6.7)	

BMI, body mass index.

Data are presented as mean ± standard deviation or counts (percentage).

*p < .05, indicates a significant difference between groups.

**Table 2:** **Intra- and postoperative data**

	**Bone resorption (n = 29)**	**Nobone resorption (n = 15)**	**p**
**Intraoperative**			
Operating time, h	2.3 ± 0.4	2.3 ± 0.5	.598
Blood loss, mL	181.4 ± 169.1	127.3 ± 85.7	.167
**Postoperative**			
Hospital stay, d	18.1 ± 3.1	16.3 ± 2.6	.058
Time wearing brace, mo			.720
≤3	11 (37.9)	8 (53.3)	
4-5	3 (10.3)	1 (6.7)	
≥6	15 (51.7)	6 (40.0)	
Time performing low back muscle exercises, mo			.299
0	13 (44.8)	8 (53.3)	
≤3	6 (20.7)	0 (0.0)	
3-6	5 (17.2)	4 (26.7)	
≥7	5 (17.2)	3 (20.0)	

Data are presented as mean ± standard deviation or counts (percentage).

L4 and L5 spinous process height, length, and thickness data are shown in Table 3. There were no significant differences in L4 or L5 spinous process height, length, or thickness at baseline and 1 week after surgery between the two groups. The L4 and L5 spinous process measurement at 1 year after surgery were significantly lower than that at 1 week after surgery in both groups (all, p ≤ .002). However, the length of the L4 and L5 spinous processes in the bone resorption group were significantly lower than that in the no bone resorption group at 1 year after surgery (both, p < .05). The frequencies of significant resorption were significantly different between the two groups with respect to the length of the L4 spinous process, and the height, length, and thickness of the L5 spinous process (all, p ≤ .008), respectively.

**Table 3:** **L4 and L5 spinous process parameters at baseline 1 week and 1 year after surgery**

	**Bone resorption (n = 29)**	**Nobone resorption (n = 15)**	**p**
**L4 spinous process**			
Height			
Baseline	83.0 ± 9.7	81.8 ± 8.7	.698
1 week	77.5 ± 9.3	76.6 ± 11.0	.776
1 year	71.2 ± 11.2	69.4 ± 11.9	.635
Significant resorption^a^	5 (17.2)	0 (0.0)	.149
Length			
Baseline	54.6 ± 7.4	57.2 ± 7.4	.279
1 week	50.5 ± 8.3	51.7 ± 8.0	.654
1 year	40.4 ± 10.1	46.7 ± 8.1	.042*
Significant resorption^a^	17 (58.6)	0 (0.0)	.001*
Thickness			
Baseline	13.2 ± 2.7	13.1 ± 2.2	.871
1 week	12.4 ± 2.6	12.1 ± 2.7	.725
1 year	11.1 ± 2.5	11.5 ± 2.7	.604
Significant resorption^a^	7 (24.1)	0 (0.0)	.077
**L5 spinous process**			
Height			
Baseline	73.9 ± 11.2	70.3 ± 11.8	.319
1 week	67.6 ± 11.3	64.3 ± 10.9	.358
1 year	56.0 ± 11.5	57.4 ± 10.4	.699
Significant resorption^a^	11 (37.9)	0 (0.0)	.008*
Length			
Baseline	42.5 ± 10.6	46.1 ± 7.6	.244
1 week	37.3 ± 10.2	41.4 ± 6.6	.167
1 year	25.6 ± 6.9	37.2 ± 7.3	<.001*
Significant resorption^a^	21 (72.4)	0 (0.0)	<.001*
Thickness			
Baseline	12.6 ± 2.9	11.4 ± 2.7	.182
1 week	11.9 ± 2.8	10.3 ± 2.8	.094
1 year	10.0 ± 2.1	9.7 ± 2.9	.704
Significant resorption^a^	12 (41.4)	0 (0.0)	.003*

Data are presented as mean ± standard deviation or counts (percentage).

*p < .05, indicates a significant difference between groups.

^a^Bone resorption rate = (value at 1 week postoperative – value at 1 year postoperative)/value at 1 year postoperative × 100. Significant resorption defined as a bone resorption rate > 20%.

There were no significant differences between the bone resorption and no bone resorption groups in the improvement rate of VAS pain score, ODI, and JOA score at 1 year after surgery, and there was no difference in MRI Pfirrmann grade between groups at 1 year after surgery (Table 4).

**Table 4:** **Symptom improvement and MRI finding at 1 year after surgery**

	**Bone resorption (n = 29)**	**Nobone resorption (n = 15)**	**p**
VAS pain score			
Baseline	8.0 (6.0, 9.0)	7.0 (6.0, 9.0)	.319
1 year	1.0 (0.0, 1.0)	1.0 (0.0, 1.0)	.957
Improvement rate^a^, %	88.9 (85.7, 100.0)	88.9 (80.0, 100.0)	.639
ODI			
Baseline	82.0 (64.0, 88.0)	70.0 (62.0, 82.0)	.224
1 year	4.0 (2.0, 8.0)	4.0 (2.0, 6.0)	.869
Improvement rate^a^, %	94.7 (90.0, 96.7)	93.5 (91.4, 96.3)	.776
JOA score			
Baseline	3.0 (2.0, 4.0)	5.0 (2.0, 5.0)	.090
1 year	14.0 (13.0, 14.0)	14.0 (13.0, 15.0)	.378
Improvement rate^a^, %	76.9 (71.4, 84.6)	66.7 (64.3, 80.0)	.164
MRI			
Baseline	4.0 (3.0, 4.0)	4.0 (3.0, 5.0)	.449
1 year	5.0 (4.0, 5.0)	5.0 (4.0, 6.0)	.786

Data are presented as median (IRQ; interquartile range).

VAS, visual analogue scale; ODI, Oswestry Disability Index; JOA, Japanese Orthopedic Association; MRI, magnetic resonance imaging.

^a^Improvement rate = (value at baseline– value at 1 year postoperative)/baseline value × 100.

Univariate analysis of risk factors associated with bone resorption showed that lumbar lordosis was significantly associated with bone resorption (Table 5). Lumbar lordosis ≥ 50° was associated with a lower rate of bone resorption than lumbar lordosis < 50° (OR = 0.14, p = .006). Multivariate analysis, after adjusting for gender, BMI, and length of hospital stay, showed that lumbar lordosis ≥ 50° was associated with a lower rate of bone resorption than lumbar lordosis < 50° (OR = 0.15, p = .035). After adjusting for gender, lumbar lordosis, and hospital stay, the odds of bone resorption increased with every kg/m^2^ increase in BMI (OR = 1.31, p = .038) (Table 6).

**Table 5:** **Univariate analysis of risk factors associated with bone resorption**

	**OR (95% CI)**	**p**
Age, y	1 (0.96,1.05)	.926
Gender		
Female	1	
Male	0.3 (0.08,1.15)	.079
BMI, kg/m^2^	1.22 (0.99,1.49)	.056
Smoking		
Never smoked	1	
Light smokers	0.63 (0.12,3.3)	.589
Severe smokers	0.29 (0.05,1.57)	.150
Osteoporosis	NA	
Lumbar lordosis,		
<50	1	
≥50	0.14(0.03,0.57)	.006*
L4-5 spinous height		
≤20	1	
20-30	2.57 (0.25,26.24)	.426
30-40	0.36 (0.08,1.64)	.186
≥40	0.86 (0.12,6.01)	.877
L4-5 interspinous height		
≤25	1	
25-30	0.38 (0.06,2.53)	.318
30-35	0.52 (0.08,3.36)	.496
≥35	0.86 (0.05,13.48)	.913
Operating time, h	1.46 (0.37,5.82)	.589
Blood loss, mL	1 (1,1.01)	.261
Hospital stay, d	1.25 (0.98,1.6)	.067
Time wearing brace, mo		
≤3	1	
4-5	2.18 (0.19,25.01)	.531
≥6	1.82 (0.49,6.76)	.372
Time performing low back muscle exercises, mo		
0	1	
≤3	NA	
3-6	0.77 (0.16,3.74)	.745
≥7	1.03 (0.19,5.51)	.976

BMI, body mass index; OR, odds ratio; CI, confidence interval.

*p <0.05, indicates significantly associated with bone resorption.

**Table 6:** **Multivariate analysis of risk factors associated with bone resorption**

	**OR (95% CI)**	**p**
Gender		
Female	1	
Male	2.9( 0.45,18.79)	.264
BMI, kg/m^2^	1.31 (1.02,1.7)	.038*
Lumbar lordosis,		
<50	1	
≥50	0.15 (0.03,0.87)	.035*
Hospital stay, d	1.24 (0.91,1.68)	.174

BMI, body mass index; OR, odds ratio; CI, confidence interval.

*p < 0.05, indicates significantly associated with bone resorption.

## Discussion

This study found that lumbar lordosis ≥ 50° was associated with a lower rate of bone resorption than lumbar lordosis < 50° and increasing BMI was associated with an increased rate of bone resorption after implantation of the Wallis dynamic stabilization device.

The Wallis system is an interspinous elastic internal fixation device that is made of PEEK and is anchored to the spinous processes by Dacron tapes [6]. The device, similar to other dynamic interspinous devices, limits lumbar spine extension and preserves lumbar spine movement to a certain degree [6,12,22]. The Wallis device is indicated for mild lumbar stenosis, large disc herniation, recurrent disc herniation, and Modic type 1 changes with low back pain [6]. The Wallis device provides good short-, medium-, and long-term outcomes and decrease the occurrence of ASD [7,8,23,24]. It also lead to disc rehydration in certain case [23]. However, recent systematic reviews of the literature found there is no clinical data from comparative studies that support the use of dynamic stabilization devices over standard fusion techniques [25], and address whether they may potential benefit a select group of patients with degenerative disease of the lumbar spine [26].

### Stress and spinous process bone resorption

Though good moderate- to long-term outcomes of the Wallis system have been reported [7,8], there is a difference between the elastic modulus of the Wallis system and that of cortical bone. After being implanted, there is a change in the stress distribution of the spine and the device, in particular, impacts the spinous process itself [27]. Moreover, application of the tension band significantly increases the stress of the contact surface between the spinous process and the implant. In addition, the device cannot correct deficiencies in the anterior and middle columns, which leads to uneven distribution of stress loading, lumbar spine instability, and even disease recurrence [11,12,27].

Significant bone resorption occurred in 29 of 44 patients (65.9%) in the current study during a 1-year follow-up period. Comparison between data obtained 1 week after surgery and that obtained 1 year after surgery showed varying degrees of bone resorption affected the length, height, and thickness of the spinous processes, and the most significantly affected were the heights of the L4 and L5 spinous process. We consider this to be related to the anatomical features of the spinous process itself, and the inherent characteristics of the Wallis elastic fixation device. The spinous process is a relatively weak structure serving as the attachment of many back muscles. It stabilizes the lumbar spine, and plays an important role in leveraging stress. However, the spinous process does not share the axial stress of the anterior and middle columns of the spine. After implantation of the Wallis system, this changes and the spinous processes of the operated segment bears some axial stress. The Wallis system is relatively rigid and can be compressed only mildly. Though the device allows flexion and extension of the lumbar spine, it increases stress loading on the spinous process [12,27]. Even with proper placement, motion between the bone and the PEEK material occurs which can cause abrasion to the bone. In addition, the tension on the band used to fix the device is considerable which further increases stress in the contact surface between the spinous process and the implant.

In 1970, Justus and Luft [28] suggested a mechanochemical hypothesis for bone remodeling induced by mechanical stress. In 1989, Chiba et al. [29] suggested that osteoclasts play an important role during bone remodeling, and that the mechanism of osteoclast differentiation and the inflammatory mechanism are different. In 1990, Tanne et al. [30] reported that bone resorption was related to traction and compressive stress, there was a positive correlation between the degree of stress and the degree of deformation at the midpoint of the bone, and that mechanical stress could result in bone resorption and bone remodeling. At the same time, Takuma et al. [31] reported that mechanical stress could induce varying degrees of bone resorption and bone remodeling. The results of these studies also support that hypothesis increased stress on the spinous process after implantation of the Wallis system leads to bone resorption around the implant. Although we did not directly measure stress, based on the concept of the three column spine model [32], we propose that increased BMI may indirectly contribute to axial stress. This idea is supported by an earlier studies that found placement of interspinous spacer and increased BMI may contribute to increased stress and bone resorption [33].

### Pattern of spinous process bone resorption

The results of this study suggest a pattern to bone resorption occurring after placement of the Wallis device. Due to the downward conduction of stress in the lumbar spine [32,34], the force is greatest in the L5 spinous process (and greater than that on the L4 spinous process). In this study, bone resorption of the L5 spinous process was greater than that of the L4 spinous process. There were more females than males in the bone resorption group (16/29, 55.2%) and bone resorption of the spinous process occurred in all patients with osteoporosis (*n* = 7). It is possible that the presence of osteoporosis was the reason why there were a greater number of females than males that experienced bone resorption. However, this analysis was not part of the current study and deserves future investigation.

### Risk factors of spinous process bone resorption

Multivariate analysis indicated that lumbar lordosis and BMI were related to the occurrence of bone resorption. A larger BMI suggests an increased load on the lumbar spine [34]. In a normal spine, this load is transmitted to the inferior segment along the anterior and middle columns. After implantation of an interspinous internal fixation device, this load is shared by the device to a certain extent, which results in an increased stress on the spinous process [12,27].

Lumbar lordosis ≥ 50° was associated with a lower rate of bone resorption than lumbar lordosis < 50° (OR = 0.15, p = .035). A change in lumbar lordosis indirectly reflects the state of stress sharing in the lumbar spine. When the angle becomes large, the stress shared by the middle and posterior columns of the spine increases, the interspinous distance may be reduced significantly, and pseudarthrosis formation may occur in some patients (which suggests increased stress in the spinous process).

This finding may in part be affected by the presence of osteoporosis. In theory, the stress-bearing ability of the trabecula is reduced in the presence of osteoporosis, the balance between bone resorption and reconstruction is affected, and stress-induced bone resorption occurs [35,36].

In theory, a certain degree of interspinous elastic device loosening may occur after bone resorption, which can result in a mild decrease of the lumbar stability and subsequent postoperative pain and discomfort due to the stimulation of inflammatory factors produced during friction and bone resorption [37]. However, the results of the study showed no association between the bone resorption and ODI, VAS pain score, JOA score, and MRI findings. The possible reason is that the follow-up period of 1-year was too short for differences between the 2 groups to become evident. Interestingly, Sobottke et al. [26] studied patients who had received either the X Stop®, Diam®, or Wallis implant and found that although there was loss of correction as determined by radiographic measurements reverting towards initial values VAS pain scores did not change.

There are limitations of this study that should be considered. The number of patients was relatively small and the follow-up period of 1 year is too short to adequately assess functional outcomes. Radiographs were used to measure bone resorption, and although efforts were taken to minimize measurement errors there is error that is unavoidable due to the inherent limitations of radiography. Computed tomography may have provided more accurate determination of bone resorption. We cannot find supporting reference papers to fully support our hypothesis that the pressure of the Wallis device against the spinous processes may contribute bone resorption. In addition, the female population was not homogenous and included women that had and had not gone through menopause. Another limitation of the study is that the L4 and L5 spinous processes were trimmed which may have affected the bone mineral density at the spinous processes. The study was designed to investigate the overall activity of the lumbar spine, hence we only measured the L1-S1 distal lordosis. It is possible that other spinal measurments would have given additional insight into the problem.

## Conclusions

Significant bone resorption occurs within 1 year after implantation of the Wallis dynamic stabilization device in more than 50% of patients. While the bone loss did not affect short-term functional results, it may be a factor in postoperative medium- or long-term pain and postoperative disease recurrence. Further follow-up is necessary to determine the long-term effect of bone loss after Wallis device implantation.

## Abbreviations

ASD: Adjacent segment degeneration
BMI: Body mass index
CIs: Confidence intervals (CIs)
IPSs: Interspinous process spacers (IPSs)
JOA: Japanese Orthopedic Association
MRI: Magnetic resonance imaging
ODI: Oswestry Disability Index
Ors: Odds ratios
PACS: Picture archiving and communication system
PEEK: Polyetheretherketone
VAS: Visual analogue scale
